# Rhomboid protein 2 of *Eimeria maxima* provided partial protection against infection by homologous species

**DOI:** 10.1186/s13567-020-00886-7

**Published:** 2021-02-18

**Authors:** Yufeng Chen, Di Tian, Lixin Xu, Ruofeng Yan, Xiangrui Li, Muhammad Ali A. Shah, Xiaokai Song

**Affiliations:** 1 MOE Joint International Research Laboratory of Animal Health and Food Safety, College of Veterinary Medicine, Nanjing Agricultural University, Nanjing, 210095 China; 2 Nanjing Ringpai Vet Hospital Co., Ltd., Nanjing, 210012 China; 3 Department of Pathobiology, PMAS Arid Agriculture University, Rawalpindi, 41000 Pakistan

**Keywords:** Rhomboid-like protein 2, *Eimeria maxima*, Immunogenicity, Protective efficacy

## Abstract

Rhomboid-like proteases (ROMs) are considered as new candidate antigens for developing new-generation vaccines due to their important role involved in the invasion of apicomplexan protozoa. In prior works, we obtained a ROM2 sequence of *Eimeria maxima* (EmROM2). This study was conducted to evaluate the immunogenicity and protective efficacy of EmROM2 recombinant protein (rEmROM2) and EmROM2 DNA (pVAX1-EmROM2) against infection by *Eimeria maxima* (*E. maxima*). Firstly, Western blot assay was conducted to analyze the immunogenicity of rEmROM2. The result showed that rEmROM2 was recognized by chicken anti-*E. maxima* serum. Reverse transcription-polymerase chain reaction (RT-PCR) and Western blot assay revealed apparent transcription and expression of EmROM2 at the injection site. qRT-PCR (quantitative real-time PCR), flow cytometry and indirect ELISA indicated that vaccination with rEmROM2 or EmROM2 DNA significantly upregulated the transcription level of cytokines (IFN-γ, IL-2, IL-4, IL-10, IL-17, TGF-β and TNF SF15), the proportion of CD8^+^ and CD4^+^ T lymphocytes and serum IgG antibody response. Ultimately, a vaccination-challenge trial was performed to evaluate the protective efficacy of rEmROM2 and pVAX1-EmROM2 against *E. maxima*. The result revealed that vaccination with rEmROM2 or pVAX1-EmROM2 significantly alleviated enteric lesions, weight loss, and reduced oocyst output caused by challenge infection of *E. maxima*, and provided anticoccidial index (ACI) of more than 160, indicating partial protection against *E. maxima*. In summary, vaccination with rEmROM2 or pVAX1-EmROM2 activated notable humoral and cell-mediated immunity and provided partial protection against *E. maxima*. These results demonstrated that EmROM2 protein and DNA are promising vaccine candidates against *E. maxima* infection.

## Introduction

Avian coccidiosis, a kind of intestinal parasitic protozoa disease, seriously impairs the poultry industry worldwide with a loss of beyond $3 billion USD per year [1]. Currently, controlling of this disease mainly depends on the usage of anticoccidial drugs worldwide [2, 3]. However, extensive drug use has caused drug resistance and drug residues in food which aroused widespread concern [4, 5]. Live vaccines are the main alternative control strategy to chemical prophylaxis [2]. Nevertheless, the conventional live vaccines are costly, not fully efficient and have a risk of diffusing pathogens [6, 7]. Vaccination with new generation vaccines including DNA vaccine and subunit vaccine is a promising strategy alternative to conventional treatments [6, 8]. For developing new generation vaccines, it is important to identify protective antigens. Thus, research efforts have been put in to find novel vaccine targets over the past several decades [9].

A series of specialized parasite molecules are required when apicomplexan protozoa entry into the host cell [10]. Rhomboid-like proteases (ROMs) are involved in the process of invasion by apicomplexan parasites [11]. Invasion by majority of apicomplexan parasites are carried out by adhesins, which mediate binding to the receptor molecules of host cell. ROMs are thought to participate in the cleavage of adhesins from receptors of host cell and result in a final entry into the host cell [12, 13]. Therefore, ROMs can be regarded as new candidate antigens for developing new-generation vaccines [13, 14].

ROMs family have been identified in apicomplexan protozoa, such as *Plasmodium* spp., *Toxoplasma gondii*, *Theileria annulata* and *Theileria parva* [13, 15, 16]. As for *Eimeria* species, the protective efficacy of ROMs (such as rFPV-rhomboid, rBCG pMV261-Rho and rBCG pMV361-Rho) from *Eimeria tenella* (*E. tenella*) has been evaluated [9, 17, 18]. Up to now, however, there are still few reports concerning the immunogenicity and protective efficacy of *E. maxima* ROM2.

In prior works, we obtained a ROM2 sequence of *E. maxima* which is the homologous gene with ROM2 of *Toxoplasma gondii*. In this study, the recombinant protein and eukaryotic expression plasmid of EmROM2 were used as subunit vaccine and DNA vaccine. Meanwhile, the immunogenicity and protective efficacy induced by EmROM2 were evaluated. Our results provide a promising vaccine candidate antigen against *E. maxima*.

## Materials and methods

### Plasmids, parasites, and animals

The prokaryotic expression vector pET-32a and eukaryotic expression vector pVAX1 were bought from Novagen (Darmstadt, Germany) and Invitrogen (Carlsbad, California, U.S.A), respectively. The oocysts of *E. maxima* were derived from our laboratory, propagating, harvesting and sporulating were carried out by the method previously described [19]. New-hatched Hy-Line chickens were raised in strictly sterilized animal house. Food and water without anticoccidial drugs were provided ad libitum. SD rats (180–200 g) were purchased from Qinglongshan Breeding Farm in Nanjing. Animal experiments were approved by the Committee on Experimental Animal Welfare and Ethics of Nanjing Agricultural University (Approval number: PAT2020001).

### Cloning of EmROM2 and construction of recombinant plasmids of pET-32a-EmROM2 and pVAX1-EmROM2

Micro glass balls was used to break the sporulated oocysts of *E. maxima* by whirl mix [19]. Total RNA of *E. maxima* sporozoites was extracted by an E.Z.N.A.™ Total RNA Kit I (OMEGA, Norcross, Georgia, U.S.A) following the product instruction. Then, HiScript II Q RT SuperMix (Vazyme, Nanjing, China) was utilized to generate the cDNAs. RT-PCR was conducted to amplify EmROM2 gene using the specific primers (Table 1). Finally, the PCR products were cloned into prokaryotic expression vector pET-32a and eukaryotic expression vector pVAX1 to create recombinant plasmids pET-32a-EmROM2 and pVAX1-EmROM2 respectively. Concisely, EmROM2 gene and pET-32a vector were cleaved by *Bam*H I and *Hin*d III, while EmROM2 gene and pVAX1 vector were cleaved by *Bam*H I and *Eco*R I, and finally ligated at the same enzyme sites. Double-enzyme digestion and sequencing were conducted to verify the recombinant plasmids.

**Table 1:** **Primers used for the construction of pET-32a-EmROM2 and pVAX1-EmROM2.**

Plasmids	Primers
pET-32a-EmROM2	Forward: 5′-CGGATCCATGGCGCGGGTTCATACTT-3′
	Reverse: 5′-CCAAGCTTTCAGGCGCAACTACGGGGGAG-3′
pVAX1-EmROM2	Forward: 5′-CGCGGATCCATGGCGCGGGTTCATACTT-3′
	Reverse: 5′-CCGGAATTCTCAGGCGCAACTACGGGGGAG-3′

### Preparation of rEmROM2 and anti-rEmROM2 serum

The expression plasmid of pET-32a-EmROM2 was transformed into the *E.coli* BL21 (DE3) to express rEmROM2, which was purified using a protein purification kit of His Trap™ FF (GE Healthcare, U.S.A). Then a ToxinEraser™ Endotoxin Removal Kit (Genscript, Nanjing, China) was used to remove the endotoxin to eliminate possible interference. Sodium dodecyl sulfate-polyacrylamide gel electrophoresis (SDS-PAGE) was performed to detect the purified rEmROM2. The rat anti-rEmROM2 serum was prepared by the protocol previously described [8] for Western blot detection. Meanwhile, the serum from non-injected rat was used as a negative control.

### Western blot recognition of rEmROM2 by chicken anti-*E. maxima* serum

Chicken anti-*E. maxima* serum was obtained by the method previously described [8, 20]. The above chicken antiserum was used as primary antibody (serum from uninfected chicken was set as negative control), and horseradish peroxidase (HRP)-conjugated goat anti-chicken IgG (Sigma-Aldrich, Darmstadt, Germany) was used as secondary antibody to carry out Western blot assay. Briefly, rEmROM2 was separated through SDS-PAGE, and next transferred to a nitrocellulose membrane (Merck millipore, Darmstadt, Germany). Subsequently, the membrane was blocked with 5% bovine serum albumin (BSA) (Takara Biomedical Technology, Dalian, China) in PBST (phosphate buffered saline-Tween) (20 mM Tris–HCl, 150 mM NaCl, 0.05% (V/V) Tween 20) overnight at 4 ℃, afterwards successively incubated with chicken anti-*E. maxima* serum (1: 100) and goat anti-chicken IgG (1:4500). Finally, 3, 30-diaminobenzidine (DAB) was used to detect the bound antibody [8].

### Detection of transcription and expression of the pVAX1-EmROM2 at the injection site through RT-PCR and Western blot

Fourteen-day-old healthy chickens were divided into two groups at random and vaccinated with 100 µg of pVAX1-EmROM2 and 100 µg of pVAX1 by intramuscular injection of leg, respectively. The pVAX1-injected muscle and non-injected muscle were set as empty vector and negative controls in transcription detection of pVAX1-EmROM2. One week later, muscle samples were collected from the pVAX1-EmROM2-injected, pVAX1-injected and non-injected sites. After grinding in a mortar, total RNA of muscle tissue was extracted using RNAiso Plus (Takara Biomedical Technology, Dalian, China) following the product instruction. Then the residual recombinant plasmid was removed by digestion with DNase I (Takara Biomedical Technology, Dalian, China) to eliminate possible interference. RT-PCR was performed to detect the transcription of EmROM2 gene with the RNA product as template using the specific primers for EmROM2 gene (Table 1). In expression detection of pVAX1-EmROM2, RIPA solution (Thermo Scientific, Waltham, MA, U.S.A) was used to treat the pVAX1-EmROM2-injected muscles for 2 h. Then Western blot assay was performed with the supernatant collected. The rat anti-rEmROM2 serum was used as primary antibody (the non-injected rat serum was set as negative control), and HRP-conjugated anti-rat IgG (Sigma-Aldrich, Darmstadt, Germany) was used as secondary antibody to detect the expression of pVAX1-EmROM2 at the injected muscle [8].

### Immunogenicity analysis of EmROM2

#### Experimental design

Fourteen-day-old healthy chickens were divided into six groups (30 chickens per group) at random. In experimental groups, 200 µg of rEmROM2 and 100 µg of pVAX1-EmROM2 were injected into the leg muscles separately. Simultaneously, 200 µg of pET-32a tag protein and 100 µg of pVAX1 were injected in tag protein control and vector control groups, respectively. Sterile PBS (phosphate buffered saline) was injected in PBS control groups. When the chickens were 21 days old, a booster vaccination was conducted as described above.

#### Flow cytometry analysis of splenic T lymphocytes subsets

One week after the primary and booster vaccination, the proportion of CD8^+^ and CD4^+^ T lymphocytes was detected with the spleens of five chickens from each group. Splenic lymphocytes were collected according to the previous protocol [21]. Under dark conditions, lymphocytes (1 × 10^7^ cells/mL) were incubated with mouse anti-chicken CD3^+^ and mouse anti-chicken CD8^+^, mouse anti-chicken CD3^+^ and mouse anti-chicken CD4^+^ (SouthernBiotech, Birmingham, AL, U.S.A) for 45 min at 4 ℃, respectively. Then PBS was used to wash the cells twice by centrifugation (2500 rpm, 3 min, 4 ℃). T lymphocytes subsets analysis was conducted using BD FACScan flow cytometer (BD Biosciences, Franklin Lakes, NJ, U.S.A).

#### Detection of cytokines transcription through quantitative real-time PCR

Total RNA of splenic lymphocytes was extracted by an E.Z.N.A.™ Total RNA Kit I (OMEGA, Norcross, Georgia, U.S.A) following the product instruction. Subsequently, cDNAs of lymphocytes were generated using HiScript II Q RT SuperMix (Vazyme, Nanjing, China) for qRT-PCR assay following the manufacturer's instruction. Specific primers for cytokines of IFN-γ, IL-2, IL-4, IL-10, IL-17, TGF-β and TNF SF15 were designed by NCBI and Primer Ques Tool (IDT). GAPDH gene was designed as internal reference control (Table 2). Meanwhile, amplification efficiencies were evaluated according to the protocol previously reported [22]. qRT-PCR was employed to determine the above mRNA level according to the instruction of ChamQ™ SYBR qPCR Master Mix Kit (Vazyme, Nanjing, China). Determination of cytokines transcription was performed using the 7500 Real Time PCR System (Applied Biosystems, Carlsbad, CA, U.S.A) with a particular program in accordance with the instruction. The fold change of the transcriptional level of cytokines was determined utilizing 2^−ΔΔCT^ method compared to the internal reference control gene of GAPDH [23].

**Table 2:** **Primers used for the quantitative real-time PCR.**

RNA target	Primers sequence	Accession NO	Amplification efficiency (%)^a^	Correlation coefficients (*r*^2^)
GAPDH	Forward: 5′-GGTGGTGCTAAGCGTGTTAT-3′	K01458	100.74	0.9917
	Reverse: 5′-ACCTCTGTCATCTCTCCACA-3′			
IL-2	Forward: 5′-TAACTGGGACACTGCCATGA-3′	AF000631	102.44	0.9921
	Reverse: 5′-GATGATAGAGATGCTCCATAAGCTG-3′			
IL-4	Forward: 5′-ACCCAGGGCATCCAGAAG-3′	AJ621735	99.09	0.9936
	Reverse: 5′-CAGTGCCGGCAAGAAGTT-3′			
IL-10	Forward: 5′-GGAGCTGAGGGTGAAGTTTGA-3′	AJ621614	99.19	0.9923
	Reverse: 5′-GAAGCGCAGCATCTCTGACA-3′			
IL-17	Forward: 5′-ACCTTCCCATGTGCAGAAAT-3′	EF570583	100.24	0.9940
	Reverse: 5′-GAGAACTGCCTTGCCTAACA-3′			
IFN-γ	Forward: 5′-AGCTGACGGTGGACCTATTATT-3′	Y07922	103.07	0.9868
	Reverse: 5′-GGCTTTGCGCTGGATTC-3′			
TGF-β	Forward: 5′-CGGGACGGATGAGAAGAAC-3′	M31160	102.79	0.9815
	Reverse: 5′-CGGCCCACGTAGTAAATGAT-3′			
TNF SF15	Forward: 5′-GCTTGGCCTTTACCAAGAAC-3′	NM_001024578	100.57	0.9930
	Reverse: 5′-GGAAAGTGACCTGAGCATAGA-3′			

^a^ Amplification efficiency (%) = (10^−1/slope^ − 1) × 100%.

#### Detection of serum IgG antibody level

Blood samples were gathered from the chickens 1 week after the primary and booster vaccination. Serum IgG antibody level was determined using indirect ELISA (enzyme-linked immunosorbant assay) [20]. In brief, 0.05 M carbonate buffer was used to dilute the purified rEmROM2 with a concentration of 10 ng/μL. A 96-well microtiter plate (Corning-Costar NY, U.S.A) was coated with 150 µL of above recombinant protein per well overnight at 4 ℃. Subsequently, the plate was blocked with 4.5% skimmed milk in PBST, and successively incubated with the chicken serum sample (1:100) and goat anti-chicken IgG antibody (Sigma-Aldrich, Darmstadt, Germany) (1:4500). Each well was incubated with 3,3,5,5-tetramethylbenzidine (TMB) (Sigma-Aldrich, Darmstadt, Germany) to develop the color. 2 M H_2_SO_4_ was used to stop the reaction. Finally, a microplate reader (Thermo Scientific, Waltham, MA, U.S.A) was utilized to detect the absorbance.

### Protective efficacy evaluation of EmROM2 against homologous infection in chickens

As shown in Table 3, 14-day-old chickens were weighed and divided into eight groups (30 chickens per group) at random. The experimental groups were intramuscularly vaccinated with 200 µg of rEmROM2 and 100 µg of pVAX1-EmROM2, respectively. Meanwhile, the tag protein control and vector control groups were injected with 200 µg of pET-32a tag protein and 100 µg of pVAX1 plasmid as the same method as the experimental group, respectively. The challenged control groups and unchallenged control groups were injected with 200 µL of sterile PBS. When the chickens were 21 days old, a booster vaccination was conducted as described above. At the age of 28 days, all the chickens, with the exception of the unchallenged control groups, were orally challenged with the sporulated oocysts of *E. maxima* (1 × 10^5^/chicken) [24]. One week later, weighting and slaughtering the chickens were performed. Finally, the protective efficacy of rEmROM2 and pVAX1-EmROM2 was evaluated based on the average body weight gain, oocyst output, enteric lesion and ACI (anti-coccidial index) [25–28].

**Table 3:** **Protective efficacy of rEmROM2 and pVAX1-EmROM2 against challenge with** ***E. maxima.***

Groups	Average body weight gain (g)	Relative body weight gain (%)	Mean lesion scores	Average OPG (× 10^5^)	Oocyst decrease ratio (%)	ACI
rEmROM2	50.25 ± 6.33^b^	88.30^b^	1.67 ± 0.65^b^	0.51 ± 0.68^b^	73.33^b^	170.60
pET-32a control	29.46 ± 11.25^c^	51.77^c^	2.66 ± 0.93^c^	2.15 ± 0.97^c^	4.44^c^	85.17
Challenged control	27.21 ± 8.52^c^	47.81^c^	2.84 ± 0.88^c^	2.25 ± 0.94^c^	0^c^	79.41
Unchallenged control	56.91 ± 10.24^a^	100^a^	0 ± 0^a^	0 ± 0^a^	100^a^	200
pVAX1-EmROM2	63.84 ± 8.80^b^	80.54^b^	1.68 ± 0.99^b^	0.57 ± 0.31^b^	79.72^b^	162.74
pVAX1 control	38.19 ± 15.39^c^	48.15^c^	2.75 ± 0.62^c^	2.80 ± 0.16^c^	0.36^c^	80.65
Challenged control	39.28 ± 9.72^c^	49.53^c^	2.83 ± 0.72^c^	2.81 ± 0.13^c^	0^c^	81.23
Unchallenged control	79.32 ± 9.59^a^	100^a^	0 ± 0^a^	0 ± 0^a^	100^a^	200

Significant difference (*p* < 0.05) between data was annotated with different letters in the same column. No significant difference (*p* > 0.05) between data was annotated with the same letter in the same column.

### Statistical analysis

One-way analysis of variance (ANOVA) was used to analyze the data obtained in accordance with Duncan's multiple range test at a 5% level by using SPSS 20 Data Editor (SPSS Inc., Chicago, IL, U.S.A).

## Results

### Cloning of EmROM2 and construction of recombinant plasmids of pET-32a-EmROM2 and pVAX1-EmROM2

**Figure 1:**
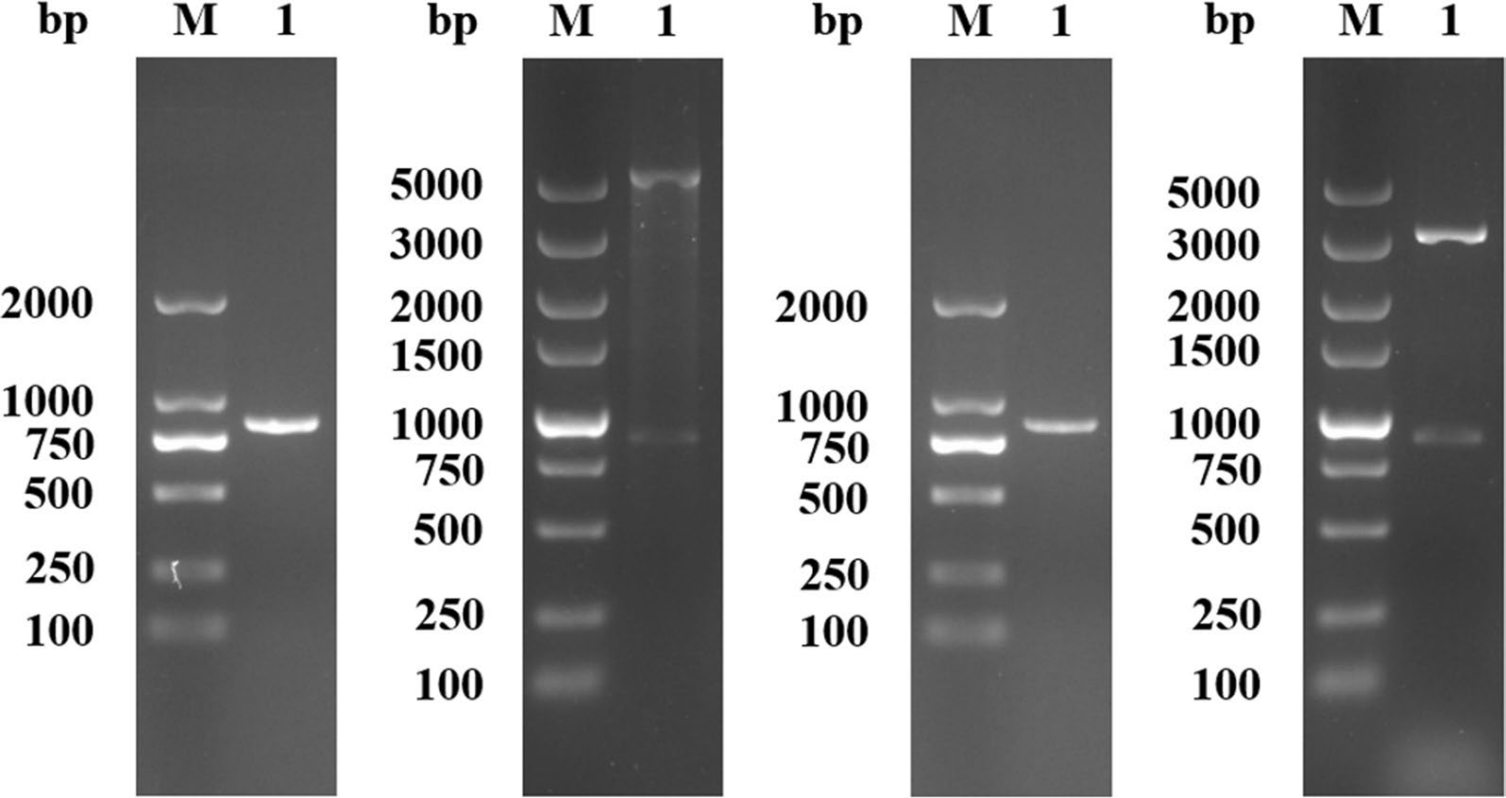
**Amplification of EmROM2 gene and identification of recombinant plasmids pET-32a-EmROM2 and pVAX1-EmROM2. A** Amplification of EmROM2 gene by RT-PCR; lane M: DL 2000 DNA marker; lane 1: amplification product of EmROM2 gene for constructing pET-32a-EmROM2; **B** Double enzyme digestion identification of pET-32a-EmROM2; lane M: DL 2000 DNA marker; lane 1: pET-32a-EmROM2 digested by *Bam*H I and *Hin*d III; **C** Amplification of EmROM2 gene by RT-PCR; lane M: DL 2000 DNA marker; lane 1: amplification product of EmROM2 gene for constructing pVAX1-EmROM2; **D** Double enzyme digestion identification of pVAX1-EmROM2; lane M: DL 5000 DNA marker; lane 1: pVAX1-EmROM2 digested by *Bam*H I and *Eco*R I.

PCR product of EmROM2 gene was detected by electrophoresis and sequencing analysis. As shown in Figure 1, electrophoresis revealed a band of 849 bp, which is equal to the target gene. Sequencing analysis revealed that the EmROM2 gene shared 100% similarity in nucleotide sequence with the gene in GenBank (Sequence ID: XM_013480878.1). Endonuclease digestion and sequencing were performed to verify the constructed recombinant plasmids of pET-32a-EmROM2 and pVAX1-EmROM2. Digestion of pET-32a-EmROM2 with *Bam*H I and *Hin*d III generated a band about 849 bp, which is equal to EmROM2 gene, and a larger band of pET-32a vector (Figure 1B). After digestion with *Bam*H I and *Eco*R I, pVAX1-EmROM2 generated a band about 849 bp of the EmROM2 gene, and a larger band, which is the linearized pVAX1 vector (Figure 1D). The sequencing analysis also verified the recombinant plasmids.

### Expression of rEmROM2 and Western blot recognition of rEmROM2 by chicken anti-*E. maxima* serum

**Figure 2:**
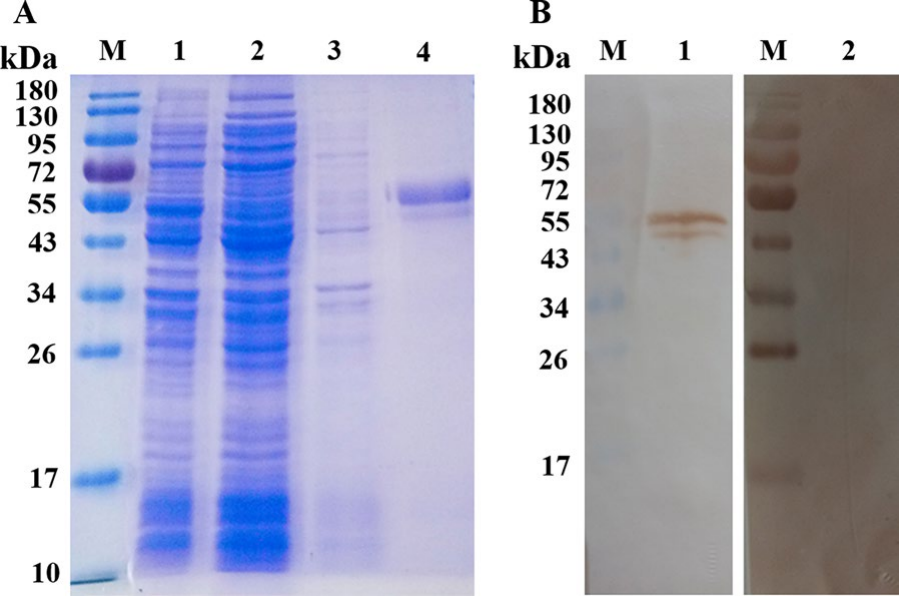
**Purification of rEmROM2 and Western blot recognition of rEmROM2 by chicken anti-E. maxima serum. A** Purification of rEmROM2; lane M: standard protein molecular weight marker; lane 1: pET-32a-EmROM2 bacterial lysate; lane 2: the supernatant of pET-32a-EmROM2 bacterial lysate; lane 3: the precipitation of pET-32a-EmROM2 bacterial lysate; lane 4: purified rEmROM2; **B** Western blot recognition of rEmROM2 by chicken anti-*E. maxima* serum; lane M: standard protein molecular weight marker; lane 1: rEmROM2 recognized by chicken anti-*E. maxima* serum; lane 2: rEmROM2 recognized by negative chicken serum.

SDS-PAGE revealed that rEmROM2 was expressed and well purified, showing a band of approximately 49 kDa which is in accordance with the predicted molecular consist of EmROM2 and pET-32a tag protein (Figure 2A, lane 4). SDS-PAGE analysis on the expression of pET-32a-EmROM2 in different time was showed in Additional file 1. Western blot assay indicated that rEmROM2 was identified by chicken anti-*E. maxima* serum (Figure 2B, lane 1).

### Transcription and expression detection of recombinant plasmid pVAX1-EmROM2 at the injection site

**Figure 3:**
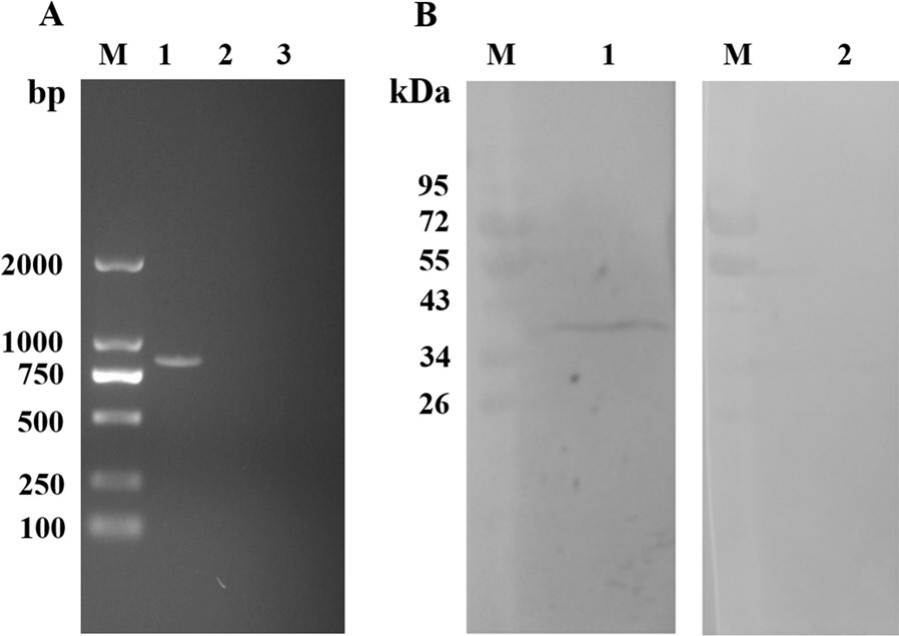
**Transcription and expression detection of recombinant plasmid pVAX1-EmROM2 at the injection site. A** Transcription detection of pVAX1-EmROM2 by RT-PCR; lane M: DL 2000 DNA marker; lane 1: product of EmROM2 from pVAX1-EmROM2-injected muscle; lane 2: pVAX1-injected muscle control; lane 3: non-injected muscle control; **B** Expression detection of pVAX1-EmROM2 by Western blot; lane M: standard protein molecular weight marker; lane 1: pVAX1-EmROM2-injected muscle recognized by rat anti-rEmROM2 serum; lane 2: pVAX1-EmROM2-injected muscle recognized by negative rat serum.

The transcription of pVAX1-EmROM2 in the injected muscles was detected through RT-PCR. As shown in Figure 3, a band about 849 bp was detected from the muscles injected with pVAX1-EmROM2 by agarose electrophoresis (Figure 3A, lane 1), indicating the transcription of pVAX1-EmROM2 at the injected muscle. Meanwhile, no band was found from non-injected muscle and pVAX1-injected muscle (Figure 3A, lanes 2 and 3). Western blot assay was conducted to detect the expression of pVAX1-EmROM2 from the injected muscles. The result revealed a single reaction band in the pVAX1-EmROM2 injected group (Figure 3B, lane 1), and no band was found in the negative control group (Figure 3B, lane 2).

### Immunogenicity analysis of EmROM2

#### Changes of T lymphocytes subsets induced by rEmROM2 and pVAX1-EmROM2

**Figure 4:**
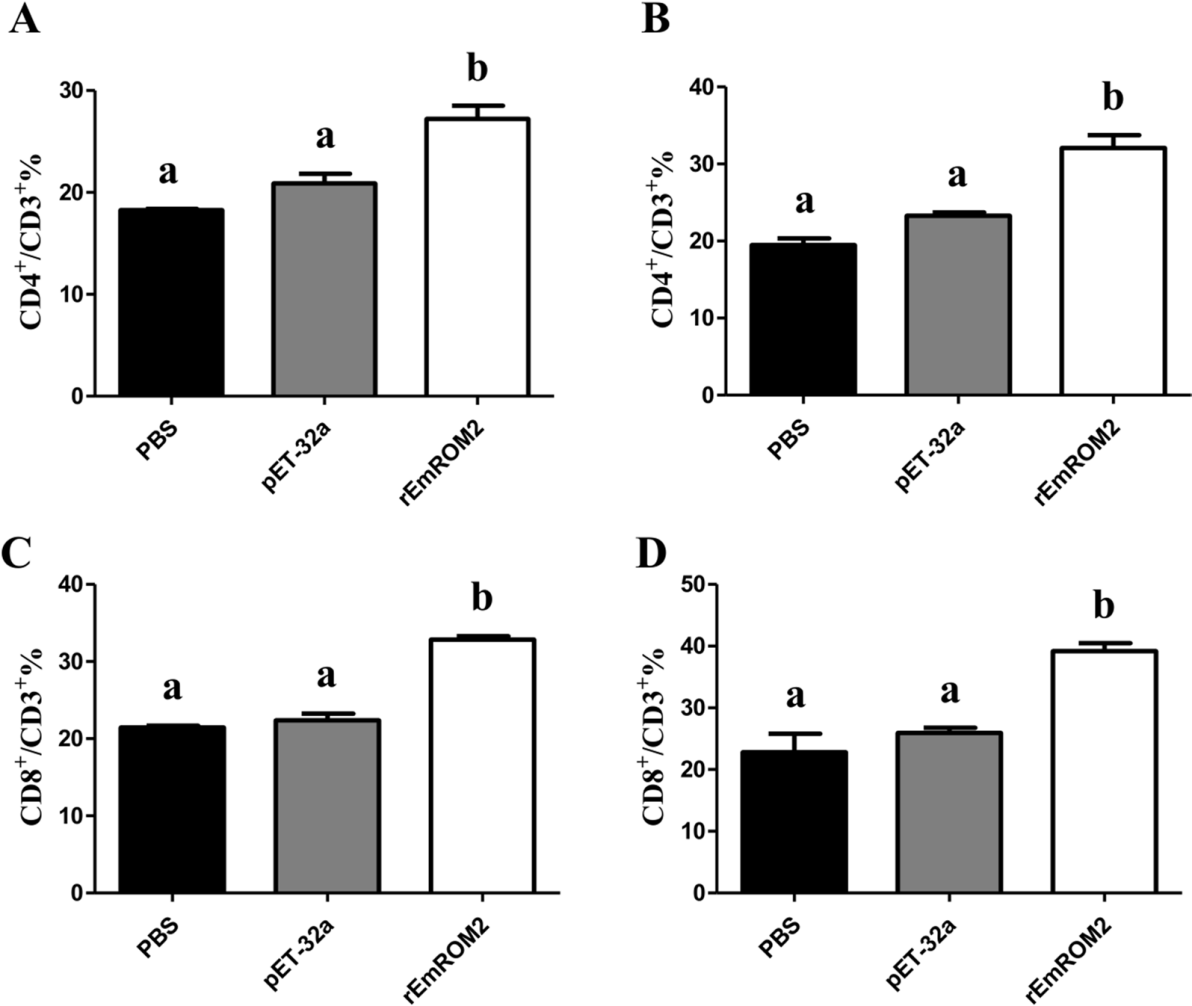
**Changes of splenic T lymphocytes subsets induced by rEmROM2 in chickens**. **A** Proportion of CD4^+^/CD3^+^ splenic T lymphocytes in chickens 1 week after the primary vaccination; **B** Proportion of CD4^+^/CD3^+^ splenic T lymphocytes in chickens 1 week after the booster vaccination; **C** Proportion of CD8^+^/CD3^+^ splenic T lymphocytes in chickens 1 week after the primary vaccination; **D** Proportion of CD8^+^/CD3^+^ splenic T lymphocytes in chickens 1 week after the booster vaccination. Significant difference (*p* < 0.05) between different groups was annotated with different letters. No significant difference (*p* > 0.05) between different groups was annotated with the same letter.

**Figure 5:**
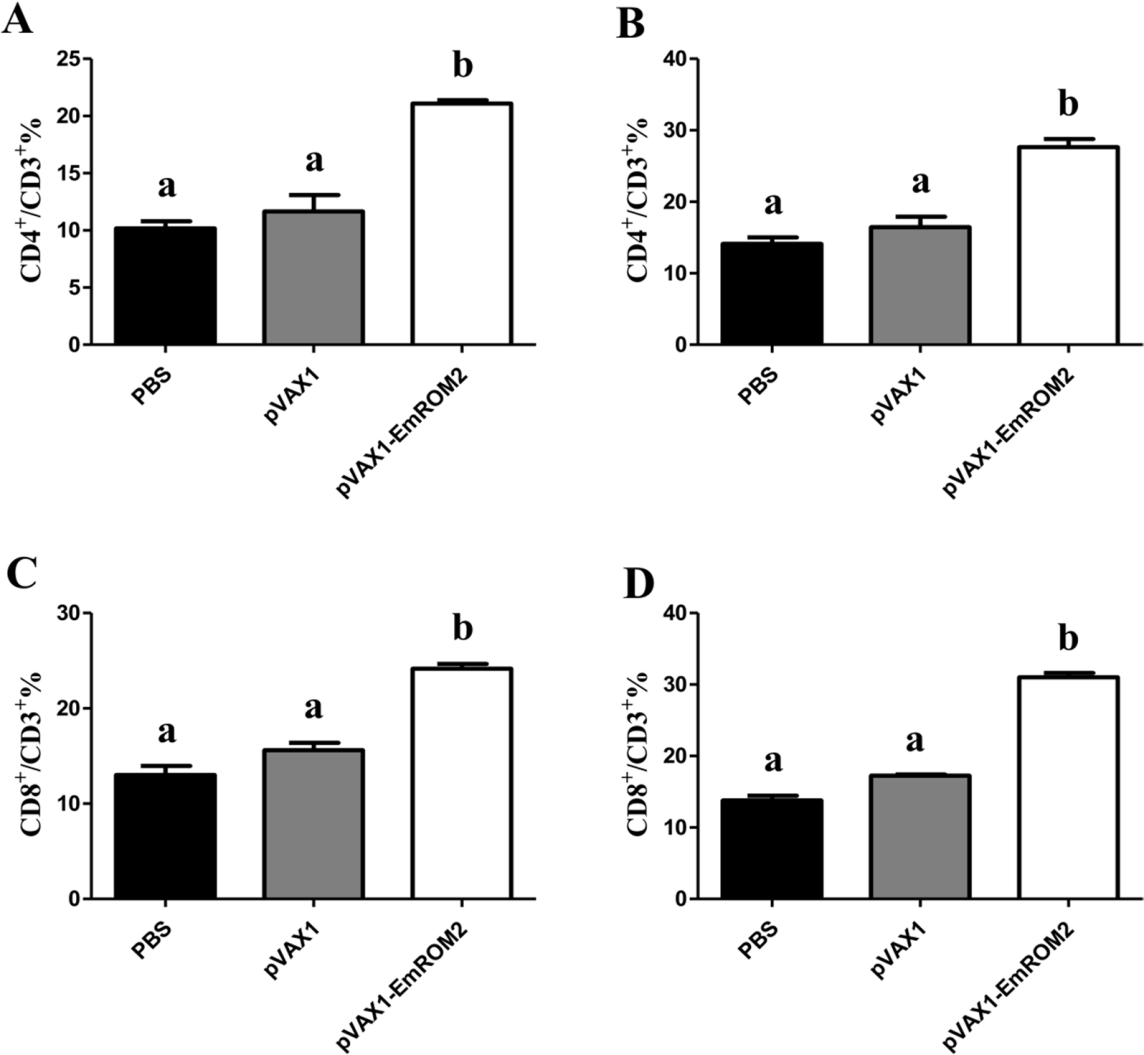
**Changes of splenic T lymphocytes subsets induced by pVAX1-EmROM2 in chickens. A** Proportion of CD4^+^/CD3^+^ splenic T lymphocytes in chickens 1 week after the primary vaccination; **B** Proportion of CD4^+^/CD3^+^ splenic T lymphocytes in chickens 1 week after the booster vaccination; **C** Proportion of CD8^+^/CD3^+^ splenic T lymphocytes in chickens 1 week after the primary vaccination; **D** Proportion of CD8^+^/CD3^+^ splenic T lymphocytes in chickens 1 week after the booster vaccination. Significant difference (*p* < 0.05) between different groups was annotated with different letters. No significant difference (*p* > 0.05) between different groups was annotated with the same letter.

Flow cytometry was performed to analyze the changes in the proportion of CD8^+^/CD3^+^ and CD4^+^/CD3^+^ T lymphocytes from the EmROM2-vaccinated chickens. The results illustrated that, by comparison with control groups, vaccination with rEmROM2 (Figure 4) or pVAX1-EmROM2 (Figure 5) obviously upregulated the proportion of CD8^+^ and CD4^+^ T lymphocytes 1 week after the primary and booster vaccination (*p* < 0.05). No notable differences were found between the pET-32a tag protein control and PBS control (*p* > 0.05), along with the pVAX1 control and PBS control (*p* > 0.05).

#### Changes of cytokines in transcriptional level induced by rEmROM2 and pVAX1-EmROM2

**Figure 6:**
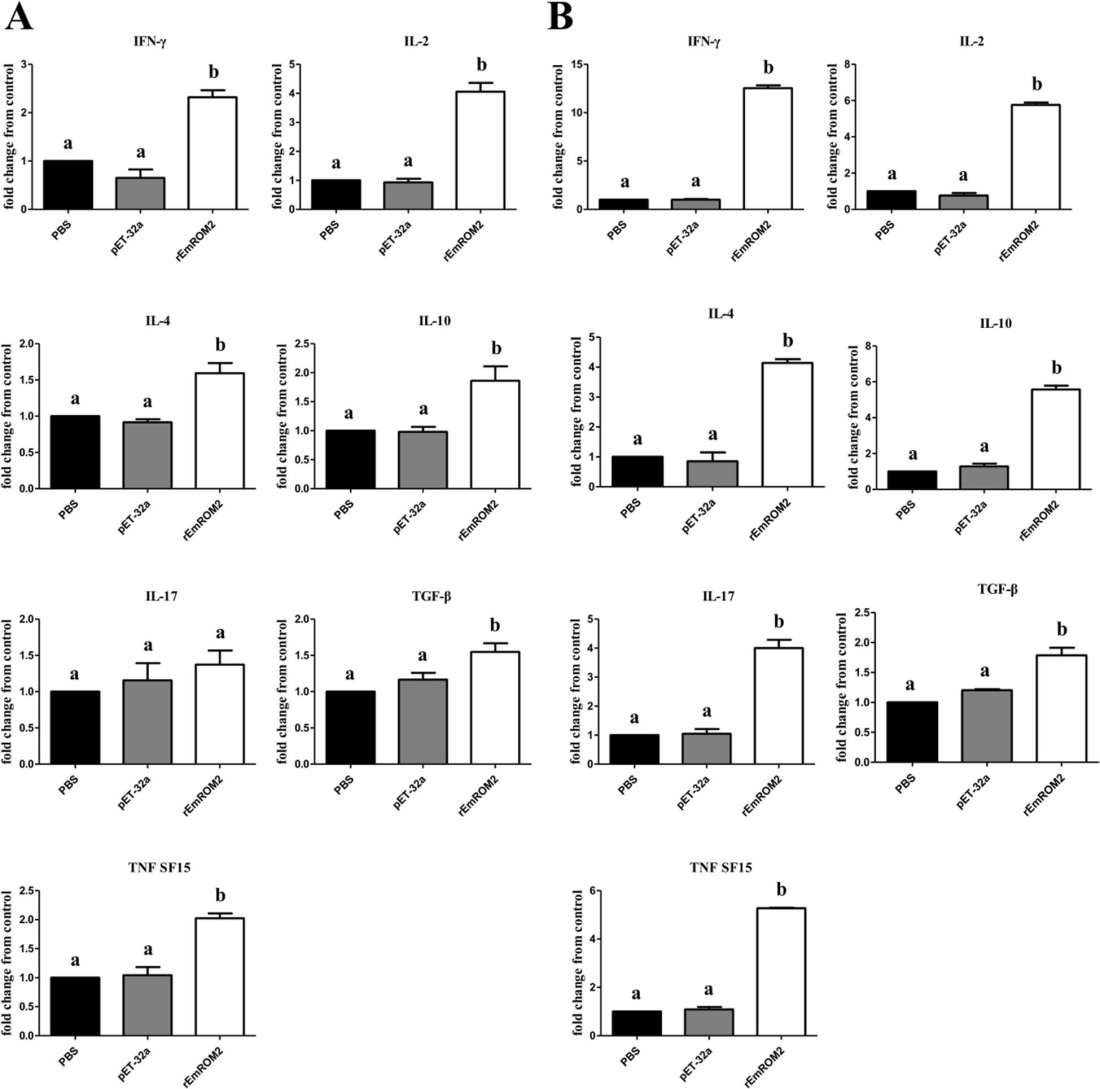
**Changes of transcriptional level of cytokines in splenic lymphocytes induced by rEmROM2. A** 1 week after the primary vaccination; **B** 1 week after the booster vaccination. Significant difference (*p* < 0.05) between different groups was annotated with different letters. No significant difference (*p* > 0.05) between different groups was annotated with the same letter.

**Figure 7:**
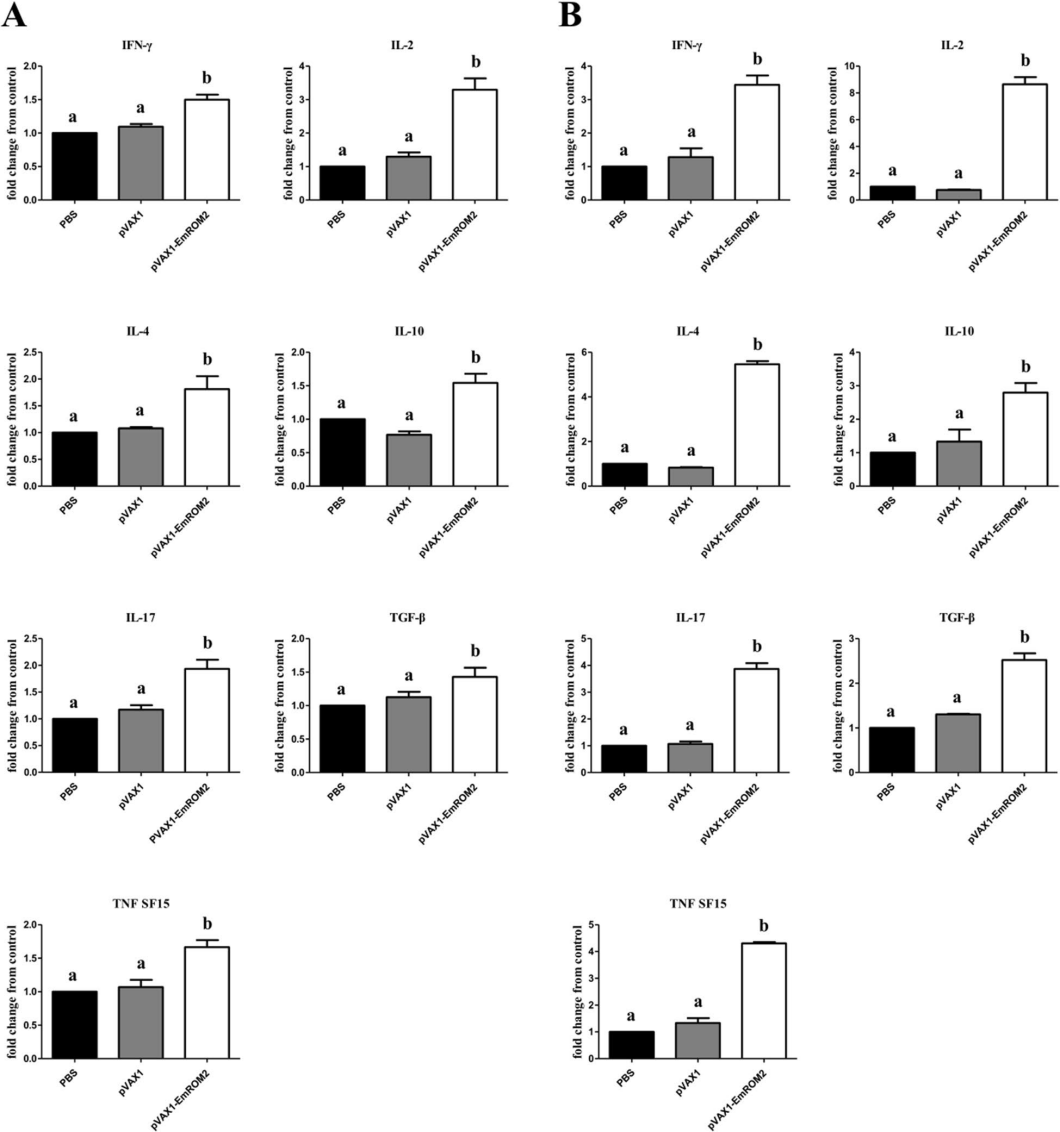
**Changes of transcriptional level of cytokines in splenic lymphocytes induced by pVAX1-EmROM2. A** 1 week after the primary vaccination; **B** 1 week after the booster vaccination. Significant difference (*p* < 0.05) between different groups was annotated with different letters. No significant difference (*p* > 0.05) between different groups was annotated with the same letter.

qRT-PCR was conducted to detect the changes in transcriptional level of IFN-γ, IL-2, IL-4, IL-10, IL-17, TGF-β4 and TNF SF15 cytokines. The rEmROM2-indcued changes in cytokines were shown in Figure 6. One week after the primary vaccination, the transcriptional level of IFN-γ, IL-2, IL-4, IL-10, TGF-β4 and TNF SF15 was obviously upregulated (*p* < 0.05), while compared to the control groups, no notable difference was found in the transcriptional level of IL-17 cytokine (*p* > 0.05). However, by comparison with the control groups, the transcriptional level of all cytokines detected was significantly increased 1 week after the booster vaccination (*p* < 0.05). No notable differences were found between the pET-32a tag protein control group and PBS control group (*p* > 0.05). In the pVAX1-EmROM2 vaccinated group (Figure 7), compared to the control groups, vaccination significantly increased the transcriptional level of all the cytokines detected 1 week after the primary and booster vaccination (*p* < 0.05). Meanwhile, no notable differences were found between the pVAX1 control group and PBS control group (*p* > 0.05).

#### Changes of serum IgG antibody level induced by rEmROM2 and pVAX1-EmROM2

**Figure 8:**
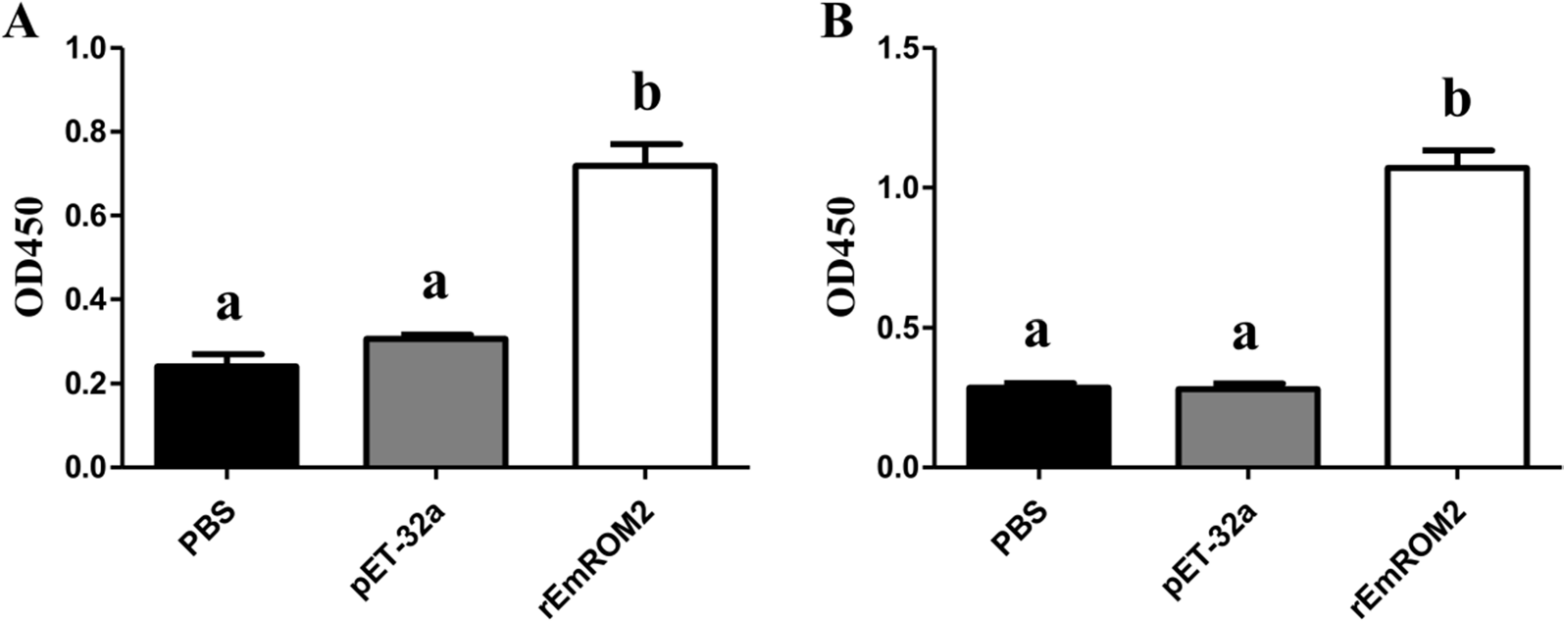
**Changes of serum IgG level induced by rEmROM2 in chickens. A** 1 week after the primary vaccination; **B** 1 week after the booster vaccination. Significant difference (*p* < 0.05) between different groups was annotated with different letters. No significant difference (*p* > 0.05) between different groups was annotated with the same letter.

**Figure 9:**
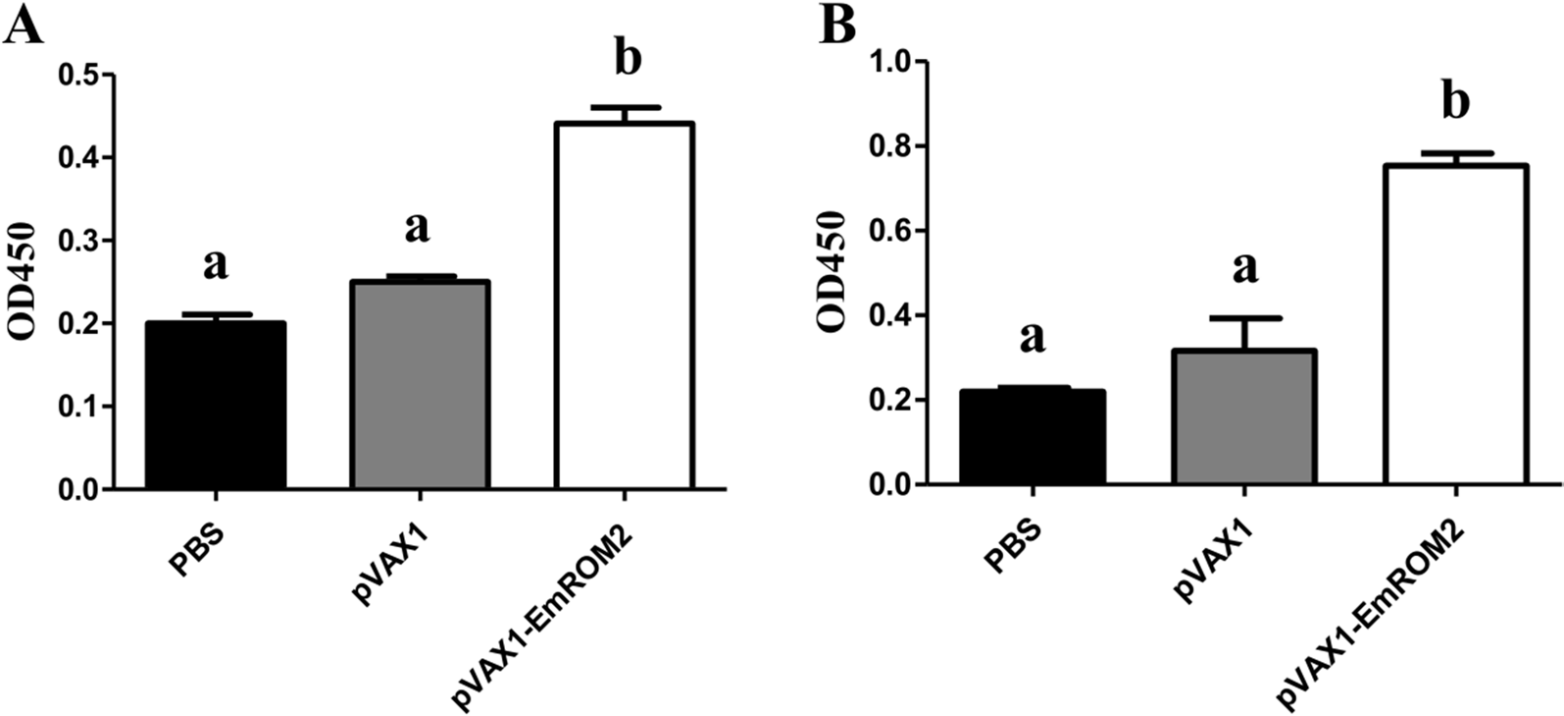
**Changes of serum IgG level induced by pVAX1-EmROM2 in chickens. A** 1 week after the primary vaccination; **B** 1 week after the booster vaccination. Significant difference (*p* < 0.05) between different groups was annotated with different letters. No significant difference (*p* > 0.05) between different groups was annotated with the same letter.

Specific serum IgG antibody level from vaccinated chickens was detected using indirect ELISA. In the rEmROM2-vaccinated group (Figure 8) and pVAX1-EmROM2 vaccinated group (Figure 9), by comparison with the control groups, vaccination significantly increased the serum IgG level from the vaccinated chickens 1 week after the primary and booster vaccination (*p* < 0.05). No notable differences were found between the pET-32a tag protein control and PBS control (*p* > 0.05), along with the pVAX1 control and PBS control (*p* > 0.05).

### Protective efficacy of rEmROM2 and pVAX1-EmROM2 against *E. maxima* infection

Protective efficacy of rEmROM2 and pVAX1-EmROM2 was evaluated via challenge with *E. maxima* (Table 3). Chickens vaccinated with rEmROM2 or pVAX1-EmROM2 significantly alleviated intestinal lesions, weight loss, and reduced oocyst output compared to chickens in challenged and pET-32a tag protein control groups (*p* < 0.05), as well as the chickens in challenged and pVAX1 control groups (*p* < 0.05). No significant differences were observed between the challenged control and pET-32a tag protein control groups (*p* > 0.05), along with the challenged control and pVAX1 control groups (*p* > 0.05). Moreover, ACI values of rEmROM2-vaccinated group and pVAX1-EmROM2-vaccinated group were 170.60 and 162.74 respectively, indicating partial protection against *E. maxima* infection.

## Discussion

As a serious intestinal disease, avian coccidiosis causes severe economic losses to the poultry industry worldwide. Vaccination with subunit or DNA vaccines is a promising alternative strategy of disease control compared to chemical prophylaxis and vaccination with live vaccines [6, 8]. In recent years, couples of antigens of *E. maxima* were tested as candidate antigens for subunit or DNA vaccines and showed promising protective efficacy, such as gam56 and gam82 [29], EmMIC2 and EmMIC7 [30, 31] and some *Eimeria* common antigens (e.g. GAPDH and 14-3-3) [8, 32]. Since ROMs are involved in the invasion of apicomplexan protozoa, they were considered as new candidate antigens for developing new-generation vaccines [13, 14]. According to the previous study, chickens vaccinated with rETRHO1 (recombinant protein of *E. tenella* rhomboid-like protein) and pVAX1-Rho (a DNA vaccine of *E. tenella* rhomboid-like protein) elicited humoral and cell-mediated immunity and generated protection against infection by *E. tenella* in chickens [14, 33]. The present study was conducted to evaluate the immunogenicity and protective efficacy of EmROM2 in forms of recombinant protein (rEmROM2) and DNA (pVAX1-EmROM2), the results showed that EmROM2 activated notable humoral and cell-mediated immunity and provided partial protection against *E. maxima*. These results demonstrated that EmROM2 protein and DNA are effective vaccine candidates against *E. maxima*.

It has been reported that cell-mediated immunity plays a major role, and humoral immunity plays a minor role in the process of immunoprotection against coccidiosis [34–36]. In the present study, the proportion of CD8^+^ and CD4^+^ T lymphocytes was obviously enhanced in the vaccinated chickens, indicating EmROM2 protein and DNA could induce cellular immune responses. Partially, cytokines could control and regulate the responses of T cells against coccidiosis. Th1-type cytokines (e.g. IFN-γ and IL-2), relating to cellular immunity, are regarded to be predominant against coccidiosis [36, 37]. IFN-γ are important in the process of regulating anticoccidial immune responses, because it can activate the phagocytosis of macrophages and killing effect of NK cells and CTLs [35]. IL-2 can induce the proliferation of T cells in vitro, and increase the proportion of CD8^+^ and CD4^+^ T lymphocytes in peripheral blood when co-delivered with vaccines in vivo [38]. In the present study, the transcriptional level of IFN-γ and IL-2 in splenic lymphocytes from chickens vaccinated with rEmROM2 and pVAX1-EmROM2 were obviously upregulated compared with the control groups (*p* < 0.05). Similarly, the transcriptional level of other five major cytokines (IL-4, IL-10, IL-17, TGF-β4 and TNF SF15) was significantly increased via the vaccination. The role of antibodies is controversial, and many reports suggest that antibodies contribute to, but are not fundamental function [39]. In some cases, however, antibodies seem to be involved in protection against coccidiosis [39, 40]. In this study, the level of specific IgG antibody was increased significantly in chickens vaccinated with rEmROM2 or pVAX1-EmROM2. In short, the changes in cytokines and IgG antibody revealed that rEmROM2 and pVAX1-EmROM2 could induce significant cellular and humoral immunity.

In this study, vaccination with EmROM2 protein or DNA significantly alleviated intestinal lesions, body-weight loss, reduced oocyst output caused by *E. maxima*. Moreover, it induced ACI values of more than 160 (170.60 and 162.74), showing partial protection against *E. maxima* infection. Furthermore, the protective efficacy could be enhanced by some methods. For example, some novel adjuvants including plant-derived adjuvants (such as saponins, and lectins) and nanoparticles (e.g. polymeric nanoparticles, inorganic nanoparticles and virus-like particles) were reported could activate or strengthen immune responses [41, 42]. Therefore, protective efficacy induced by rEmROM2 and pVAX1-EmROM2 can be augmented through co-administered with these adjuvants. Additionally, the protective efficacy of vaccines could also be improved by optimizing the vaccination route, dose, time and age of primary vaccination in chickens [43].

## Supplementary Information

**Additional file 1.** **SDS-PAGE analysis of the expression of pET-32a-EmROM2 in different time**. Lane M: standard protein molecular weight marker; lane 1: pET-32a bacterial lysate; lane 2: pET-32a induced by IPTG for 5 h; lane 3-lane 8: pET-32a-EmROM2 induced by IPTG for 0-5 h.

## Data Availability

The datasets during the current study available from the corresponding author on reasonable request.

## Abbreviations

ACI: Anti-coccidial index
bp: Base pairs
BSA: Bovine serum albumin
cDNA: Complementary DNA
Ct: Cycle threshold
DAB: 3,3′-Diaminobenzidine tetrahydrochloride
EmROM2: Rhomboid protein 2 of *Eimeria maxima*
*E. maxima*: *Eimeria maxima*
*E. tenella*: *Eimeria tenella*
ELISA: Enzyme-linked immunosorbant assay
HRP: Horseradish peroxidase
IgG: Immunoglobulin G
kDa: Kilo dalton
M: Mole/L
mRNA: Message RNA
OD: Optical density
PBS: Phosphate buffered saline
PBST: Phosphate buffered saline-Tween
PCR: Polymerase chain reaction
qRT-PCR: Quantitative real-time PCR
RIPA: Radio immunoprecipitation assay
rEmROM2: EmROM2 recombinant protein
RT-PCR: Reverse transcription-PCR
SDS-PAGE: Sodium dodecyl sulfate polyacrylamide gel electrophoresis
TMB: 3,3,5,5′-Tetramethyl benzidine
