# Supplementary figures and images for: Rhomboid protein 2 of Eimeria maxima provided partial protection against infection by homologous species
Source: Vet Res. 2021 Feb 18;52:29. doi: 10.1186/s13567-020-00886-7 (PMC7893758; doi:10.1186/s13567-020-00886-7)

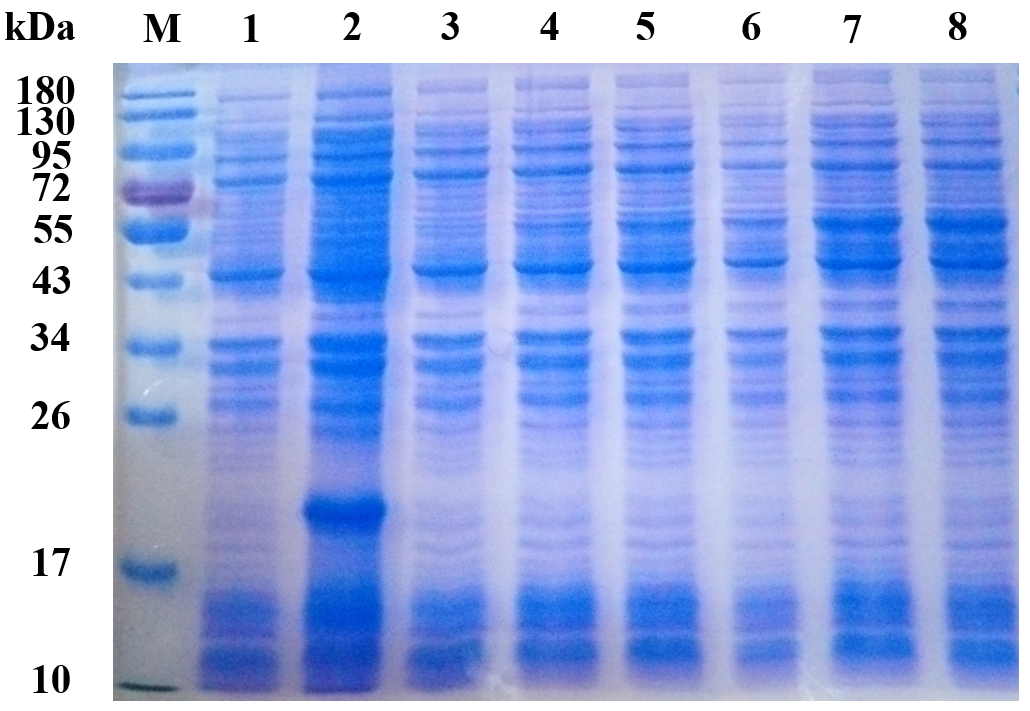

Supplement: Supplementary file 1 — Additional file 1. SDS-PAGE analysis of the expression of pET-32a-EmROM2 in different time. Lane M: standard protein molecular weight marker; lane 1: pET-32a bacterial lysate; lane 2: pET-32a induced by IPTG for 5 h; lane 3-lane 8: pET-32a-EmROM2 induced by IPTG for 0-5 h. [file 13567_2020_886_MOESM1_ESM.tif]
